# The association between demographic factors, user reported experiences and user satisfaction: results from three casualty clinics in Norway

**DOI:** 10.1186/1471-2296-11-73

**Published:** 2010-10-06

**Authors:** Kirsten Danielsen, Oyvind A Bjertnaes, Andrew Garratt, Oddvar Forland, Hilde Hestad Iversen, Steinar Hunskaar

**Affiliations:** 1 Norwegian Knowledge Centre for the Health Services, PO Box 7004 St. Olavs plass, 0130 Oslo, Norway; 2 National Centre for Emergency Primary Care, Uni Health, PO Box 7810, 5020 Bergen, Norway; 3 Haraldsplass Deaconess University College, Ulriksdal 10, 5009 Bergen, Norway; 4 Department of Public Health and Primary Health Care, University of Bergen, PO Box 7804, NO-5020 Bergen, Norway

## Abstract

**Background:**

User reported experiences and satisfaction are increasingly used as basis for quality indicators in the health sector. However, there is limited understanding of factors associated with user reported experiences and satisfaction with casualty clinics.

**Methods:**

A random sample of 542 patients that had contacted any of three casualty clinics from mid April to mid May 2008 was mailed a questionnaire. A reminder was sent to non-respondents after six weeks. Descriptive statistics for four user reported experiences scales and 20 single items are presented. Multivariate regression analysis was used to assess associations between background variables and user reported experiences, and between user reported experiences and user satisfaction.

**Results:**

225 (41.5%) patients, carers and guardians returned a completed questionnaire. Users reported most positive experiences with the doctor services and the nursing services at the casualty clinics; on a scale from 0 to 100, where 100 is the best possible experience the doctor scale was 82 and the nursing scale 81. Users reported least positive experiences with the organization of the casualty clinic, with a scale score of 65. Self perceived health was associated with user satisfaction, while self perceived health and age were associated with user reported experiences with organization of the clinics. A range of user reported experience domains were related to user satisfaction, after controlling for socio-demographic variables, including experiences with doctor services at the clinics, organization of the clinics, information and self perceived incorrect treatment.

**Conclusions:**

Users report positive experiences with the three casualty clinics, with organization as the aspect with largest improvement potential. The importance of age and health status for users' experiences and satisfaction with casualty clinics was shown, but a range of user reported experiences with the clinics were the most important predictors for user satisfaction.

## Background

Patient satisfaction and experiences are an important part in the evaluation of health care quality [1,2]. The purpose of patient satisfaction surveys is frequently related to quality improvement, but reports of general satisfaction have limited value in quality improvement processes [3,4]. Rather than simply asking about patient satisfaction, patient experiences studies identify concrete health care aspects that are important as measures of service quality from the perspective of the patients and hence contribute to their overall satisfaction. This requires an extensive development phase to secure the content validity of the questionnaire and the comparison of the domains of care included in patient experiences questionnaires with more general measures of patient satisfaction as part of validity testing. To inform quality improvement it is common to benchmark results against results for other units or changes over time. This requires case-mix analysis and potential adjustments for case-mix differences. Therefore, quality improvement based on patient satisfaction surveys requires both an assessment of the association between patient reported experiences and patient satisfaction, and between background variables and patient reported experiences of concrete aspects of health care.

A systematic review of the patient satisfaction literature showed that among socio-demographic factors, age and health status are consistently related to patient satisfaction [5]; older respondents and healthier respondents generally have higher satisfaction, while the evidence related to gender, ethnicity and socio-economic status is equivocal. The importance of age and self-perceived health for patient experiences was also found in a recent US study among community health centers, but the authors also identified other important factors like education and race/ethnicity [6]. The generalizability of these findings to the primary care out-of-hours field is uncertain. We found a few studies about the association between demographic factors and patient satisfaction with primary care out-of-hours services [7-10]. One study found that health status and socio-economic status are significantly related to patient satisfaction, but not age and gender [8], while another found that age and ethnicity are related to patient satisfaction [10]. All in all, findings are equivocal and the only pattern that emerges across these studies is that most socio-demographic factors seem to be only weakly related to patient satisfaction.

The systematic review on patient satisfaction also found consistent evidence that the most important health service factor affecting patient satisfaction is the patient-practitioner relationship [5]. Health service factors might be measured by means of patient reporting or evaluation on items and scales for patient experiences, or by other variables concerning organization and type of services. One study about the association between patient experiences and patient satisfaction relating to primary care out-of-hours services found that the doctor's assistant's attitude on the phone, opinion of GP's treatment and waiting time were strongly related to overall satisfaction [7]. Another study confirmed the importance of waiting time for patients' satisfaction [10]. The association between organization and service variables has been assessed in several primary care out-of-hours studies. Two studies found that patients receiving telephone advice were less satisfied than other patients [7,10], but other studies did not find such an association [8,9].

The studies above show that the association between socio-demographic factors and patient satisfaction are equivocal within the primary care out-of-hours field. To some degree the existing evidence contradicts findings from the general patient satisfaction literature. The literature also identifies a need for more research regarding the importance of health service factors for patients' satisfaction including factors related to patient reported experiences. Therefore, our study had two primary aims: i) to assess the association between socio-demographic factors and user reported experiences with primary care out-of-hours services; ii) to assess the association between user reported experiences and user satisfaction, controlling for socio-demographic factors.

The analyses were based on a study of three casualty clinics in Norway in 2008. Norway has a two-level public health care system with a small private sector. Four Regional Health Authorities (RHA) under the Ministry of Health and Care services have responsibility for the hospital sector. The 431 municipalities are responsible for organizing primary health care including out-of-hours services [11]. There were 262 out-of-hours districts in Norway in 2006 covering single or several municipalities through inter-municipality co-operatives. Emergency medical services are usually managed at GP offices during office hours, and by municipality maintained out-of-hours duties by GPs during evenings, nights and weekends [11]. Most of the out-of-hours services are located in a casualty clinic in the host municipality, but some use GPs' surgeries. In this paper we use the term casualty clinic when referring to the physical clinics, while primary care out-of-hours services refers to all services offered by the clinics including office visits, telephone advice and home visits.

## Methods

Three casualty clinics were chosen to represent different organization types and size of such clinics. The clinics were recruited through the Watchtower project [11]. The study population included patients that had been in contact with one of the three casualty clinics from 15 April to 13 May 2008. For patients younger than 16 years and patients not able to answer themselves their carers and guardians were asked to fill out the questionnaire. The survey included a random sample of 200 patients from each clinic having telephone contact, consultation visit or home visits by a doctor.

The casualty clinics distributed the questionnaires by mail to the patient's home address. We aimed to send out the reminders at two weeks, but due to practical circumstances relating to clinical administration and postal service delays reminders were sent after six weeks. The questionnaires were returned to the Norwegian Knowledge Centre for the Health Services.

The Norwegian Social Science Data Services approved the survey which was in accordance with the Helsinki Declaration of 1975, as revised in 1983. The leader of each casualty clinic had the opportunity to exclude individuals from the survey based on ethical considerations.

### Questionnaire and variables

The questionnaire used in this study has undergone a rigorous process of development and evaluation, including a literature review, qualitative interviews with patients, guardians and carers, and input from an expert group of out-of-hours staff [12,13]. These activities ensured the content validity of the questionnaire. Most of the questionnaire's core items had low levels of missing data, indicating the acceptability and relevance of the questions to patients and guardians. The psychometric tests showed that the questionnaire has satisfactory internal consistency and construct validity [13].

The questionnaire comprises five parts: parts A and E are completed by all patients; part B concerned telephone contact with the clinic; part C concerned consultation at the casualty clinic; part D concerned home visit from a doctor. The questionnaire comprised a total of 42 items including background questions about patients and guardians. The reference period used for survey questions was "during the last visit/encounter".

Based on factor analysis four scales were identified [13]: telephone contact (4 items), nursing services (4 items), doctor services (4 items), and organization (3 items). The questions are described in table 1. Item-scale correlations were above 0.5 for all scales. Cronbachs alpha was 0.91 for telephone contact, 0.90 for doctor services at the casualty clinic, 0.93 for nursing services at the casualty clinic, and 0.82 for organizing the casualty clinic, all of which are regarded as satisfactory [13]. Items relating to experiences of care have a five-point scale of Not at all, To a small extent, To some extent, To a large extent and To a very large extent. Items were transformed to scores ranging from 0 to 100 where 100 is the best possible. Items comprising scales are summed and transformed into percentage scores. Patients with missing values on more than half of the items in a scale were excluded.

**Table 1:** Mean scale and item scores for experiences with the casualty clinics on a 0-100 scale

Scale/item	N	Mean	SD
*Telephone contact*	148	80.6	16.4
The questions below are about the person you spoke with on the phone when you called the casualty clinic. Do you feel that this person:			
Took you seriously	147	80.3	19.2
Was interested in your problem	148	79.4	19.1
Was understandable	148	84.0	16.0
Was competent	143	79.4	19.5
*Doctor services*	186	81.9	17.6
The questions below are about the doctor(s) you met at the casualty clinic. Do you feel that he/she:			
Took you seriously	187	82.1	20.2
Was interested in your problem	183	80.7	20.8
Was understandable	185	83.9	17.5
Was competent	185	81.1	21.0
*Nursing services*	153	81.4	16.8
The questions below are about the nurse(s) you met at the casualty clinic. Do you feel that he/she:			
Cared for you	151	79.3	20.8
Took you seriously	151	80.8	19.0
Was understandable	152	84.2	15.9
Was competent	152	81.6	17.7
*Organization at the clinic*	193	64.7	25.6
Did you receive adequate information about how long you might expect to wait until you came in for an examination/treatment?	188	55.5	34.5
Do you think the time you had to wait from you arrived until you came in for an examination/treatment was acceptable?	191	66.2	31.3
Did you get the impression that the casualty clinic was well organized?	192	71.9	22.8
*Questions independent of contact type*			
Do you think you got enough information about your own condition?	215	73.4	23.7
Did you get enough information about the tests and examinations you went through?	165	71.2	26.4
Did you have any unanswered question after the contact with the casualty clinic?	212	71.2	31.1
Do you think that you in any way were incorrect treated by the casualty clinic (after what you are able to evaluate)?	211	91.4	19.8
All in all, are you satisfied with the help you got from the casualty clinic?	223	78.1	21.5

### Analysis

Descriptive statistics for respondents, the four scales and single items are presented (n, mean, SD). Multivariate regression analysis was used to assess the association between background variables and five dependent variables including four user reported experiences scales and one item about global satisfaction. Age and health have been found to be consistently related to patient satisfaction [5] and were included in the regression. We also included other background variables with some empirical evidence of an association with patient satisfaction, including education [6], gender [14,15], extent of urgency [15] and marital status [16]. Length of stay has been found to be associated with inpatient satisfaction [17], and in our study we used the number of times in contact with the out-of-hours clinic the last two years as an equivalent to this in the outpatient setting. We pooled the guardian and patient sample and included a respondent variable in the regression.

Finally, we assessed the association between user reported experiences and global satisfaction by means of a multivariate regression analysis, controlling for background variables. We controlled for the same background variables as in the first regression. The telephone and nursing scales, which were relevant to a fewer number of respondents, were excluded from this analysis due to small sample sizes. SPSS (15.1) was used to analyze the data.

## Results

### Data collection

Of the 600 patients included in the study, 58 were excluded due to cancellations and factors such as unknown addresses. Of the 542 patients who were sent a questionnaire, 225 (41.5%) responded. Table 2 shows the respondents characteristics; 148 (68.2%) were patients, 53 (24.4%) were guardians of children under the age of 16, and 16 (7.4%) were carers of patients above 16 years of age. Approximately two thirds of the respondents were women (71.7%), the average age was 46 years, and more than half of the respondents (58.1%) had first contacted the casualty clinic by telephone and then had a consultation at one of the three clinics.

**Table 2:** Respondent characteristics (n = 225)^a^

Variable	N^b^	%
Gender:		
Female	157	71.7
Male	62	28.3
Age of respondent, mean (SD)	215	46 (18.5)
Type of contact:		
Telephone only	20	9.3
Telephone and consultation visit	125	58.1
Consultation visit only	63	29.3
Telephone and home visit	4	1.9
Other	3	1.4
Health status		
Excellent	38	17.5
Very good	65	30.0
Good	54	24.9
Fairly good	43	19.8
Poor	17	7.8
Education:		
Primary school	42	19.5
Secondary school	73	34.0
University college/university (1-4 years)	67	31.2
University college/university (4 years or more)	33	15.3
Marital status:		
Married	110	51.2
Cohabitant	50	23.3
Living alone	55	25.6
Number of times in contact with the out-of-hours service two last years:		
1 time	51	23.7
2 times	53	24.7
3-5 times	82	38.1
6-10 times	18	8.4
More than 10 times	11	5.1
Extent of urgency:		
Very serious	34	15.5
Serious	96	43.8
Less serious	59	26.9
Uncertain about seriousness	30	13.7

^a ^Of the 225 respondents 148 (68.2%) were patients, 53 (24.4%) guardians of children < 16 years of age, and 16 (7.4%) carers of patients aged 16 years or older.

^b ^The number of respondents is 225, but item missing means that the number of respondents on each background factor varies.

### Statistical analysis

Table 1 shows mean scale and item scores for experiences with the casualty clinics. The users reported most positive experiences with the doctor services and nursing services at the casualty clinic. The former scale had an average score on 81.9, the latter 81.4. The telephone contact scale had a mean score of 80.6, while organization at the casualty clinic had a mean score of 64.7. The items about health care personnel being understandable and self perceived incorrect treatment had the highest scores for the individual items. The two questions about waiting time at the casualty clinic had the lowest scores.

Table 3 shows the results of multivariate regression analysis with background variables as independent variables and the four patient experiences scales and one item about global satisfaction as dependent variables. Explained variance for the regression models ranged from 5.6% (doctor services) to 11.4% (organization). Only a few background variables had a significant association with the dependent variables. Self perceived health was significantly associated with global satisfaction (p = .03), while age (p = .02) and self perceived health (p = .04) were significantly related to the scale about organization of the casualty clinic.

**Table 3:** Multivariate linear regression models: association between background variables and the five dependent variables

	Telephone contact (n = 130)		Nursing services -at the casualty clinic(n = 139)		Doctor services -at the casualty clinic (n = 164)		Organization-at the casualty clinic(n = 171)		Global satisfaction(n = 197)	
	B	Signifi-cance	B	Signifi-cance	B	Signifi-cance	B	Signifi-cance	B	Signifi-cance
Female (reference: male)	1.97	0.59	3.00	0.41	3.75	0.29	-1.78	0.72	-0.14	0.34
Age	0.08	0.51	0.15	0.23	0.07	0.57	0.36	0.02	0.01	0.19
Health status	-2.30	0.10	-1.18	0.45	-1.44	0.35	-4.42	0.04	-0.13	0.03
Education (reference: primary school)										
Secondary school	1.21	0.80	1.98	0.65	-2.35	0.58	2.39	0.68	0.03	0.86
University college/university (1-4 years)	5.51	0.20	3.86	0.38	0.93	0.83	4.48	0.45	0.34	0.07
University college/university (4 years or more)	4.11	0.46	6.85	0.20	-5.78	0.28	-0.15	0.98	0.10	0.65
Marital status (reference: married)										
Cohabitant	-1.88	0.63	1.61	0.70	-0.82	0.84	1.36	0.80	-0.08	0.62
Living alone	2.47	0.53	4.93	0.24	-0.19	0.96	-2.40	0.67	-0.04	0.81
Number of times in contact with the out-of-hours service two last years (reference: 1 time)										
2 times	2.26	0.60	3.28	0.45	0.20	0.96	3.15	0.58	0.18	0.29
3-5 times	3.06	0.46	1.72	0.69	-3.90	0.34	-5.31	0.34	0.07	0.69
6-10 times	0.16	0.98	4.67	0.45	-2.41	0.69	6.36	0.44	0.04	0.87
More than 10 times	5.12	0.45	9.49	0.17	4.38	0.55	2.30	0.82	0.43	0.18
Extent of urgency (reference: very serious)										
Serious	-5.68	0.17	-4.38	0.32	-4.21	0.35	-9.24	0.14	-0.13	0.49
Less serious	-4.41	0.34	-8.42	0.10	-2.28	0.65	-11.75	0.09	-0.27	0.20
Uncertain about seriousness	-0.08	0.99	0.20	0.97	-1.48	0.79	-0.05	1.00	0.10	0.66
Respondent group (reference: patients)										
Cares of children under the age of 16	-1.11	0.77	-7.11	0.07	-2.40	0.55	-1.66	0.76	-0.17	0.32
Cares of patients aged 16 or older	-5.13	0.36	-5.39	0.43	-5.21	0.46	-11.05	0.27	-0.23	0.37

Explained variance (R^2 ^): Telephone contact (0.077), Nursing services(0.086), Doctor services (0.056), Organization (0.114) and Global satisfaction (0.084).

Table 4 shows the results of multivariate regression analysis with user satisfaction as the dependent variable and user reported experiences as predictors, controlled for background variables. Explained variance for the regression model was 73.2%. Most patient reported experiences were significantly associated with user satisfaction, while two were close to significant (information on tests and unanswered questions). The most important predictors were doctor services (p < .00) and incorrect treatment (p < .00).

**Table 4:** Multivariate linear regression model: association between background variables, user reported experiences and global satisfaction (n = 154)

Variable	B	SD	t	Significance
*Socio-demographic factors:*				
Female (reference: male)	-0.13	0.09	-1.40	0.16
Age	-0.00	0.00	-0.53	0.60
Health status	-0.00	0.04	-0.06	0.95
Education (reference: primary school)				
Secondary school	-0.07	0.11	-0.61	0.54
University college/university (1-4 years)	0.08	0.12	0.66	0.51
University college/university (4 years or more)	-0.07	0.14	-0.54	0.59
Marital status (reference: married)				
Cohabitant	0.04	0.10	0.39	0.70
Living alone	-0.24	0.11	-2.10	0.04
Number of times in contact with the out-of-hours service two last years (reference: 1 time)				
2 times	0.06	0.11	0.59	0.56
3-5 times	-0.00	0.11	-0.03	0.98
6-10 times	-0.00	0.15	-0.02	0.98
More than 10 times	0.32	0.19	1.66	0.10
Extent of urgency (reference: very serious)				
Serious	0.03	0.12	0.23	0.82
Less serious	-0.22	0.14	-1.60	0.11
Uncertain about seriousness	0.07	0.15	0.46	0.64
Respondent group (reference: patients)				
Cares of children under the age of 16	-0.09	0.11	-0.86	0.39
Cares of patients aged 16 or older	0.52	0.21	2.52	0.01
*Patient experiences:*				
Doctor services at the casualty clinic (scale)	0.02	0.00	5.19	0.00
Organization at the casualty clinic (scale)	0.01	0.00	2.51	0.01
Information on own condition (item)	0.14	0.07	2.16	0.03
Enough information on the tests and examinations (item)	0.11	0.06	1.86	0.07
Unanswered question (item)	-0.06	0.04	-1.64	0.10
Incorrect treatment (item)	-0.23	0.06	-3.66	0.00

Explained variance global satisfaction (R^2 ^): 0.732.

## Discussion

In general, age and self perceived health are the most consistent socio-demographic factors related to patient satisfaction [5]. However, within the primary care out-of-hours field the few identified studies revealed inconsistencies [7-10]. One study found the importance of health status and socio-economic status, but not age and gender [8], another found the importance of age and ethnicity [10]. The only clear finding was that most socio-demographic factors seem to be only weakly related to patient satisfaction. Our study identified self-perceived health as a significant predictor for patient satisfaction and one of four experiences scales, and age as significant associated with user experiences with organization at the clinics. This follows the general patient satisfaction literature [5], and these variables have partial empirical support in the primary care out-of-hours literature. However, the small number of studies and lack of consistent findings means that associations should be assessed in future studies. Furthermore, to use this information in case-mix adjustments further work is necessary including assessing the variation of these variables across the unit of analysis [6]. Future studies in the primary care out-of-hours field might use these findings to test hypotheses about associations between health, age and patient satisfaction, but the effects of case-mix will depend on both the strength of association and the variation between the units in question.

The majority of the user reported experiences domains had a significant association with user satisfaction, after controlling for user characteristics. The most important predictor was the experiences the users had with the doctors at the casualty clinic. This concurs with findings from the systematic review of the patient satisfaction literature [5] and shows that the most important user experience domain for user satisfaction is the relationship between the user and the caregiver. The final regression model found that more than 70% of the variation in global satisfaction was explained, which also gives strong support to the validity of the user experiences questions as an indirect measure of user satisfaction. Since ratings of general satisfaction have limited value in quality improvement processes [3,4], the approach of asking about experiences with health care providers is used as a means to identify concrete improvement areas.

The primary aims of this paper were to assess the associations between socio-demographic variables and user satisfaction/experiences, and between user-reported experiences and global satisfaction. Naturally, the sample of three clinics is inadequate to represent the population of clinics in Norway. Also, the number of clinics is too small for multilevel regression, making it difficult to separate individual and clinic level effects. This means that individual level effects might be overestimated, especially if the intraclass correlation coefficient (ICC) is substantial. Future studies in Norway should include a representative sample of casualty clinics, and enough clinics to allow empirical assessment of effects at different levels.

The questionnaire is developed specifically for primary care out-of-hours users and includes items of relevance for measurement of user reported experiences with out-of-hours care. It is based on a literature review, interviews with users of out-of-hours services and consultation with an expert group that was designed to ensure the content validity of the questionnaire [12,13]. Compared to questionnaires such as the Clinician & Group CAHPS survey or the EUROPEP questionnaire, this questionnaire can be used with different types of contact with the out-of-ours services (telephone contact, and/or at the casualty clinic, and/or home visit from the doctor). This questionnaire is also designed for patients that have had one contact with the doctor which is in contrast to questionnaires specific to general practice care that relate to consultations with the patient's usual general practitioner.

The consideration of any differences found through the comparison of user experiences and satisfaction of out-of-hours care with those for general practice care more generally might inform quality improvement initiatives. The EUROPEP questionnaire has been used in large scale surveys of general practice in Norway [18]. However, the differences in the content of items, including items scaling, and scales composition rules out any comparison. Moreover, the study findings relate to three clinics and hence are not representative of all out-of-hours clinics.

Low response rates are a common problem in patient experience surveys in general [5], and also in the primary care out-of-hours field [7,19-22]. The response rate in our study was 41.5% which is similar to other surveys in this field [7]. A low response rate may cause non-response bias if non-respondents differ systematically from respondents [5]. Some studies have found differences on socio-demographic variables between respondents and non-respondents [5,20-22], but a Dutch study on patient satisfaction with out-of-hours primary care found that overall satisfaction did not differ much between respondents and non-respondents [7]. This corresponds to findings in studies conducted by the Norwegian Knowledge Centre for the Health Services that shows small differences between respondents and non-respondents in user experiences surveys [23-26]. Therefore, we expect small effects related to non-response in our study.

## Conclusions

Users report positive experiences with the three casualty clinics, with organization as the aspect with largest improvement potential. The importance of age and health status for users' experiences and satisfaction with casualty clinics was shown, but a range of user reported experiences with the clinics were the most important predictors for user satisfaction.

## Competing interests

The authors declare that they have no competing interests.

## Authors' contributions

All authors were involved in the development of the questionnaire. KD planned the paper together with the other authors, carried out the statistical analysis, conducted the study together with administrative staff, and drafted most of the paper. OAB planned the paper together with the other authors, wrote parts of the paper, and revised the draft critically and approved the final version. AG planned the paper together with the other authors, revised the draft critically and approved the final version. OF planned the paper together with the other authors, revised the draft critically and approved the final version. HHI planned the paper together with the other authors, revised the draft critically and approved the final version. SH planned the study together with the other authors, revised the draft critically and approved the final version. All authors read and approved the final manuscript.

## Pre-publication history

The pre-publication history for this paper can be accessed here:

http://www.biomedcentral.com/1471-2296/11/73/prepub
